# High Resolution Population Maps for Low Income Nations: Combining Land Cover and Census in East Africa

**DOI:** 10.1371/journal.pone.0001298

**Published:** 2007-12-12

**Authors:** Andrew J. Tatem, Abdisalan M. Noor, Craig von Hagen, Antonio Di Gregorio, Simon I. Hay

**Affiliations:** 1 Spatial Ecology and Epidemiology Group, Department of Zoology, University of Oxford, Oxford, United Kingdom; 2 Malaria Public Health and Epidemiology Group, Centre for Geographic Medicine, Kenya Medical Research Institute (KEMRI), University of Oxford, Wellcome Trust Collaborative Programme, Nairobi, Kenya; 3 Somali Water and Land Information Management Project, Food and Agriculture Organisation of the United Nations, Nairobi, Kenya; 4 Global Land Cover Network, Food and Agriculture Organisation of the United Nations, Rome, Italy; University of Southampton, United Kingdom

## Abstract

**Background:**

Between 2005 and 2050, the human population is forecast to grow by 2.7 billion, with the vast majority of this growth occurring in low income countries. This growth is likely to have significant social, economic and environmental impacts, and make the achievement of international development goals more difficult. The measurement, monitoring and potential mitigation of these impacts require high resolution, contemporary data on human population distributions. In low income countries, however, where the changes will be concentrated, the least information on the distribution of population exists. In this paper we investigate whether satellite imagery in combination with land cover information and census data can be used to create inexpensive, high resolution and easily-updatable settlement and population distribution maps over large areas.

**Methodology/Principal Findings:**

We examine various approaches for the production of maps of the East African region (Kenya, Uganda, Burundi, Rwanda and Tanzania) and where fine resolution census data exists, test the accuracies of map production approaches and existing population distribution products. The results show that combining high resolution census, settlement and land cover information is important in producing accurate population distribution maps.

**Conclusions:**

We find that this semi-automated population distribution mapping at unprecedented spatial resolution produces more accurate results than existing products and can be undertaken for as little as $0.01 per km^2^. The resulting population maps are a product of the Malaria Atlas Project (MAP: http://www.map.ox.ac.uk) and are freely available.

## Introduction

The global human population is growing by over 80 million a year, and though confidence intervals are large, is projected to reach the 10 billion mark within 50 years [1]. The vast majority of this growth is expected to be concentrated in low income countries, and primarily in urban areas [2]. The effects of such rapid growth are well documented, with the economies, environment and health of nations, amongst others, all undergoing significant change [3], [4].

High resolution, contemporary data on human population distributions are a prerequisite for the accurate measurement of the impacts of population growth, for monitoring changes and for planning interventions. Spatial databases of human population have found use in disease burden estimation, epidemic modelling, resource allocation, disaster management, accessibility modelling, transport and city planning, poverty mapping and environmental impact assessment amongst others [5]–[9]. Whilst high-income countries often have extensive mapping resources and expertise at their disposal to create such databases, across the low income regions of the world, relevant data are either lacking or are of poor quality. For many low income countries the last significant mapping efforts occurred in the 1960–70s. The scarcity of mapping resources and skilled personnel, lack of reliable validation data and difficulty in obtaining high resolution contemporary census statistics remain major obstacles to settlement and population mapping in these regions.

In producing maps of gridded population distribution, the principal factor affecting accuracy has been shown to be the administrative boundary level, or spatial resolution, of the input census data [10]. Ancillary data on such aspects as roads, topography and settlements can be incorporated to improve mapping accuracies, but unless these data are provided at a level of detail finer than the accompanying census data, their use is detrimental to mapping accuracy compared to the simple gridding of census data [10]. The intrinsic link between human population distribution and land cover [11], particularly settlements, means that such data offer the best opportunity for improved population mapping. Combinations of different types of medium spatial resolution satellite imagery have been shown to be capable of producing accurate, low-cost and easily updatable settlement maps over large areas [12]–[14]. Here, we investigate whether the outputs from these settlement mapping approaches can be integrated with land cover and census data to improve mapping accuracies over existing population distribution products. We focus on the East African (EA) region (Burundi, Kenya, Rwanda, Tanzania and Uganda) and aim to examine the feasibility of using simple and semi-automated methods that can be implemented with free image processing software and minimal personnel to produce easily updatable maps at 100 m spatial resolution. Given the scales and speed with which population growth and urbanisation are occurring in the region, such features are a necessity.

## Materials and Methods

An abridged, step by step version of the materials and methods is presented here. Full details are provided in Text S1.

### Satellite Imagery

Radarsat-1 country mosaics (MDA Geospatial Services, Richmond, Canada) comprising of data collected between 1999 and 2002 were processed to extract texture information [13], [14]. Landsat Enhanced Thematic Mapper (ETM) scenes covering the EA region for 2002 (or as close to this year as possible) were also acquired (Global Land Cover Facility, http://glcf.umiacs.umd.edu). Imagery choice was constrained by attempting to maintain between-scene temporal consistency [15] and minimising cloud cover and other detrimental atmospheric effects. Each scene was subject to atmospheric correction [16], [17] and georegistration against ancillary data layers. Figure S1 shows the Landsat ETM tile extents, shaded by month of acquisition, while Figure S2 shows the Radarsat mosaic for the EA region.

### Land cover and other data sources

Full resolution Africover (www.africover.org) land cover data for the EA region countries were obtained. The 99 individual classes were aggregated to a more generic 22 classes to provide a consistent legend across the entire region. Figure S3 shows the resulting map. In addition, Africover roads, rivers and towns products were obtained, along with other data on national parks, urban centres and health facilities to aid mapping, testing and accuracy assessment. The roads dataset was supplemented with various other datasets to ensure the inclusion of smaller unpaved roads [18].

### Census Data

**Figure 1:**
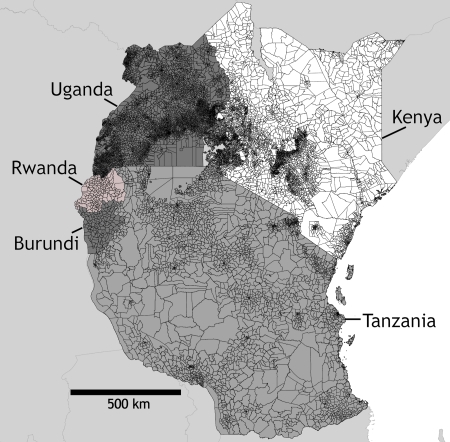
The highest levels of administrative boundaries for which national census data were available for Burundi, Kenya, Rwanda, Tanzania and Uganda.

**Figure 2:**
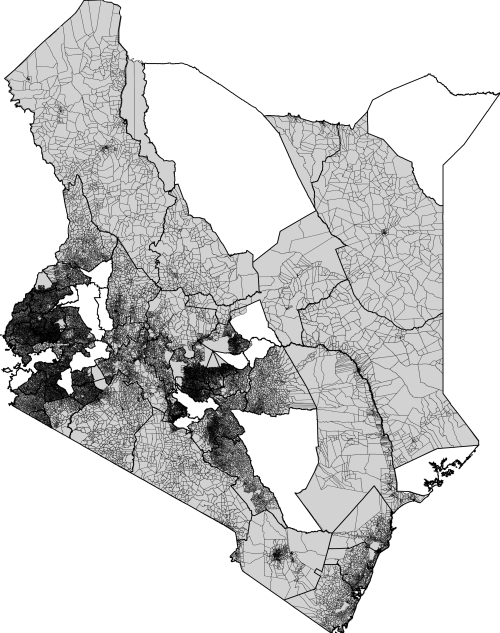
Kenya enumeration area census data. The 50 Kenyan districts for which enumeration area census data were available are shaded grey. Within each district the enumeration area boundaries are shown.

Human population census data and corresponding administrative unit boundaries at the highest level available from the most recent censuses in Burundi (1999, administrative level 2 (commune)), Kenya (1999, administrative level 5 (sublocation)), Rwanda (1991, administrative level 2 (commune)), Tanzania (2002, administrative level 4 (ward)) and Uganda (2002, administrative level 4 (parish)) were obtained (figure 1). Also obtained were Kenyan 1999 census data at enumeration area level (finer than level 5) with corresponding boundaries for 50 of the 69 Kenyan districts (figure 2). Finally, to aid population map assessment, 1999 Kenya settlement population counts were obtained and matched to corresponding Africover ‘urban area’ or ‘rural settlement’ polygons.

### Gridded population products

To enable brief comparisons of population maps produced using the approaches outlined in this paper and existing gridded population products, for Kenya the African Population Database (APD, http://www.na.unep.net/globalpop/africa/), the Global Rural-Urban Mapping Project (GRUMP, http://sedac.ciesin.org/gpw/), the Gridded Population of the World version 3 (GPW3, http://sedac.ciesin.org/gpw/) and Landscan 2005 (http://www.ornl.gov/sci/landscan/) were obtained.

### Settlement mapping

Identification of settlements and the mapping of their extents were based upon the methodologies outlined in Tatem et al [13], [14] and adapted for simplification, ease of repetition and the incorporation of new data.

Settlement mapping was undertaken at the level of the Landsat tile extent (Figure S1), with the Radarsat imagery and texture layers cut to match these extents. In areas of great topographic variation, the radar responses due to the topography are often greater than, or mistaken for, those from settlements, so a ‘terrain ruggedness index’ [19] image was created from a 90 metre spatial resolution Shuttle Radar Topography Mission (SRTM) digital elevation model (DEM). For those pixels with a value of 500 or greater (defined as ‘highly’ or ‘extremely’ rugged), Landsat ETM imagery alone was used for settlement mapping. Settlement types and reflectance characteristics are often dependent upon their setting and surrounding land cover. For the imagery in each tile extent, therefore, Africover data was used to identify land cover units (excluding settlement polygons) within which separate settlement mapping would take place. Accounting for the wide variation in reflectances within the generic land cover classes, the imagery representing each land cover type in a tile was then clustered into a conservative 1000 classes (thus reducing the possibility of spectral confusion through choosing too few classes) using ISODATA unsupervised classification [20].

Within each Landsat tile extent, 75% of Africover ‘urban area’ and ‘rural settlement’ polygons were chosen randomly for training, with the remainder set aside for accuracy assessment. For every tile, within each land cover region for which unsupervised classification had taken place, examples of ‘urban area’ and ‘rural settlement’ Africover polygons were identified from the training set, where possible. Within these polygons, image classes were highlighted and merged iteratively to best represent the training polygon extents, whilst discounting clear non-settlement land covers within the polygons, and produce a satellite imagery derived settlement map. The individual tile-level settlement maps were then mosaiced, and the overlap regions between tiles checked for consistency.

The remaining 25% of Africover settlement polygons were rasterised to the same 30 m spatial resolution grid as the settlement maps, and all grid squares within each settlement extent were identified for the calculation of accuracy statistics. An equal sized set of ‘non-settlement’ grid squares were randomly selected from non-settlement Africover classes to test whether the predicted settlement maps had identified false areas of settlement [13]. Half of these grid squares were positioned randomly within 500 m buffers of Africover settlement polygons to also assess the accuracy by which settlement extents were delineated by the maps. Percentage correct, Kappa and errors of commission and omission were calculated [21]. Visual comparison with the raw Landsat imagery and overlay onto Google Earth (where the highest resolution images were available) also enabled subjective examination of small settlement mapping accuracy where validation data did not exist.

### Population mapping

Population data for Burundi, Kenya and Rwanda were adjusted forward [10] to estimated 2002 levels using inter-censal growth rates to match the most recent census data used and the majority of the satellite imagery. Three approaches to the creation of gridded population distribution maps were tested. Firstly (EApop1), the census data was simply areal-weighted [10] to a 100 m spatial resolution grid. Secondly (EApop2), the satellite derived settlement map for each country was degraded to the same 100 m grid, and census counts within an administrative unit were then allocated to the grid squares classified as settlement. Administrative units not containing any grid squares that were classified as settlements had their population counts simply areal weighted. Finally (EApop3), the satellite imagery derived settlement maps were degraded to 100 m spatial resolution and ‘burned’ into the Africover land cover layers to create a refined land cover map for the region. Where settlement extent was mapped as smaller in the settlement map than the ‘urban area’ or ‘rural settlement’ classes in Africover, the surrounding land covers were grown to infill the gaps. This refined land cover layer and Kenyan enumeration area census data were then used to define per land cover class population densities (Table S1), were then used as weights to distribute the census data across the entire region to create a population map.

The accuracies of the various population mapping procedures were tested principally using the enumeration area level census data for 50 Kenyan districts (figure 2). Additionally, to provide a finer resolution measure of settlement population mapping accuracy, the Africover settlement extents with assigned populations were used. For each 100 m gridded population distribution map produced, the population numbers falling within each enumeration area and settlement polygon were extracted and compared against the actual population figures, with overall and district-specific root mean square errors (RMSEs) calculated. To explore the effectiveness of the population mapping procedures in the absence of high resolution census data, for Kenya the map production process was repeated using census data at administrative levels 4, 3, 2, 1 and 0 (national). Finally, the maps of Kenya from existing gridded population products (APD, GRUMP, GPW3 and Landscan 2005) were adjusted to 2002 [10], areal weighted to a 100 m grid and compared to the enumeration area census data to obtain estimates of their accuracy relative to the approaches outlined in this paper.

## Results

### Settlement mapping

**Figure 3:**
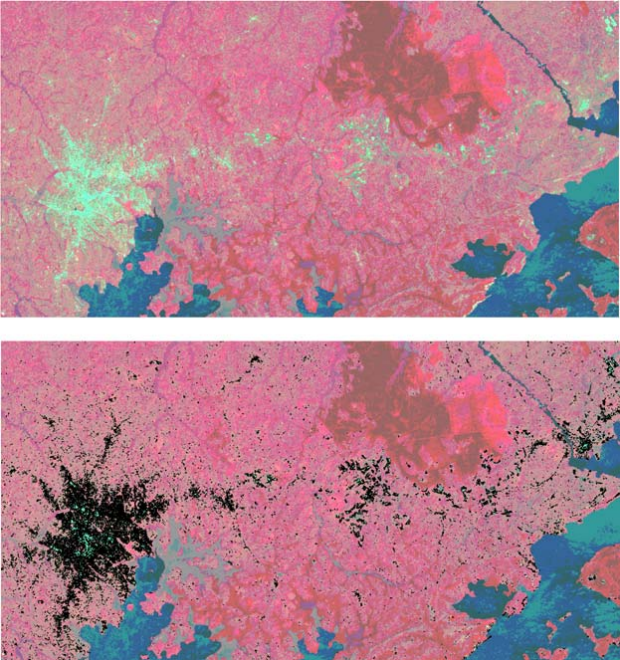
Example of settlement mapping. (a) Landsat ETM false colour composite of bands 2,3 and 4 in red, green and blue respectively showing Kampala, Uganda and surrounding areas, (b) The same image as (a), but with the outlines of mapped settlements overlaid.

Settlement maps at 30 m spatial resolution for the five EA region countries were produced from the Landsat ETM and Radarsat imagery, and figure 3 shows results for Kampala, Uganda. The results of the accuracy assessments undertaken using the randomly selected pixels, both outside and within the 25% of Africover ‘urban’ and ‘rural settlement’ polygons not used in the map production process, are in table 1. The highest overall accuracy (combined settlement and non-settlement accuracies) was 85.5% for Kenya, but results were similar across the region. In general, the non-settlement test pixels were mapped more accurately than the Africover-defined settlement pixels, although all accuracies were above 70% and Kappa values all above 0.55 (table 1), indicating a good to excellent agreement [21].

**Table 1:** Accuracy statistics for the settlement maps produced for each country in the EA region.

Country	No. Africover settlement test polygons	No. test pixels settlement/non-settlement	Settlement percentage correct	Non-settlement percentage correct	Kappa	Settlement C(%)	Settlement O(%)	Non-settlement C(%)	Non-settlement O(%)
Burundi	12	6782/6782	75.9	82.1	0.579	19.1	24.1	22.7	17.9
Kenya	81	46137/46137	81.4	89.5	0.712	11.4	18.6	17.2	10.5
Rwanda	11	6222/6222	76.8	83.9	0.607	17.3	23.2	21.6	16.1
Tanzania	147	83595/83595	73.9	92.2	0.662	9.5	26.1	22.1	7.8
Uganda	80	45585/45585	72.1	83.5	0.557	18.6	27.9	25.0	16.5

C = Error of commission, O = Error of omission.

### Population mapping

**Figure 4:**
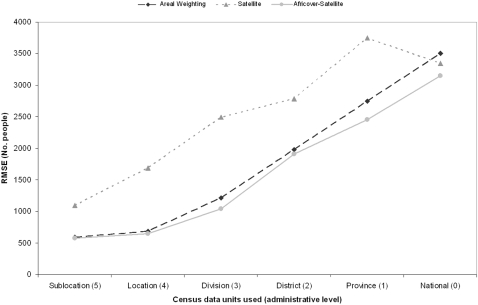
RMSE plots of the three population mapping procedures tested for the six different administrative levels of Kenyan census data.

**Figure 5:**
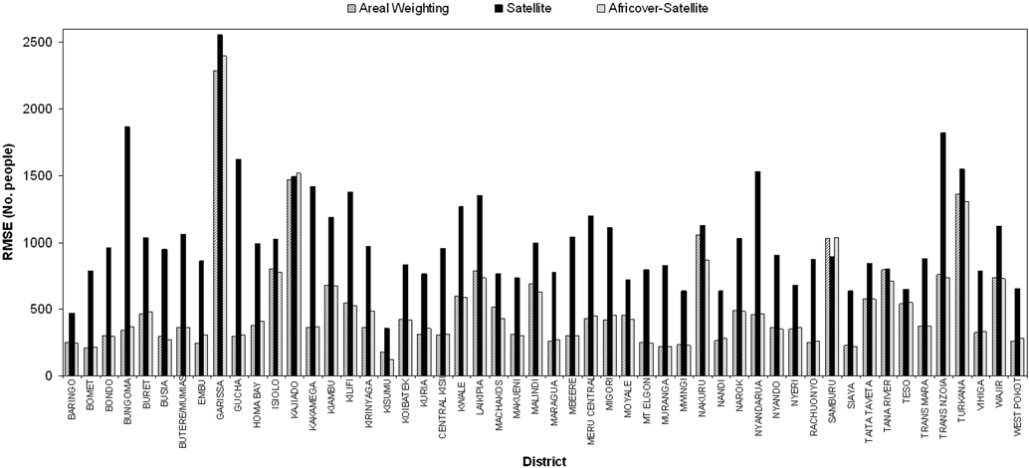
Per-district RMSE plots of the three population mapping procedures.

**Figure 6:**
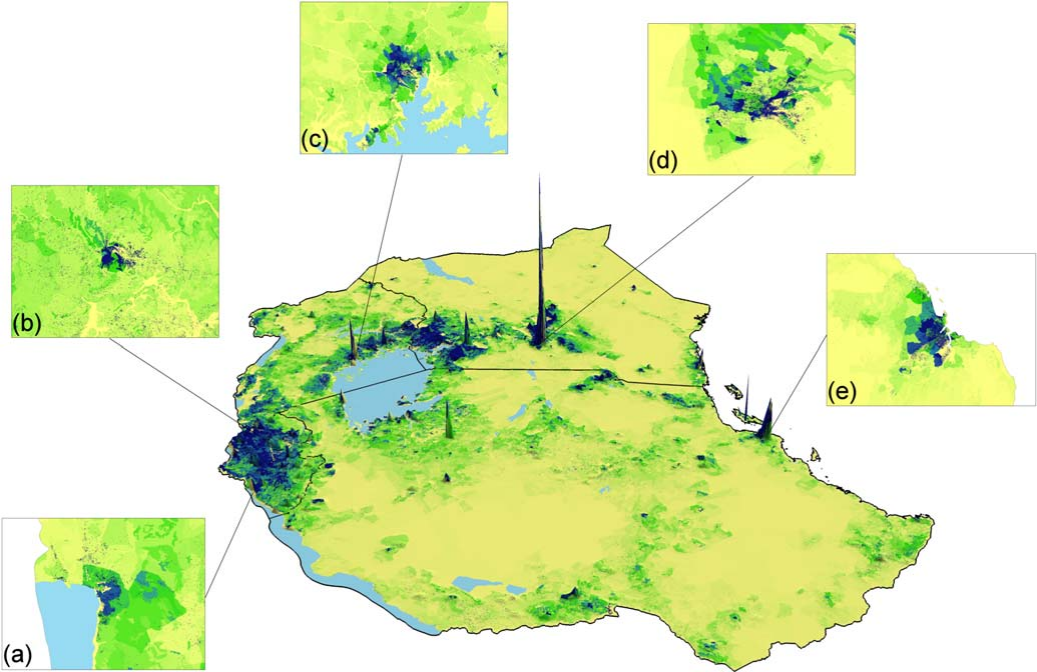
East Africa region population density estimated using the EApop3 approach. The spatial resolution has been degraded and vertical exaggeration has been applied for visualisation purposes. The full-resolution close-ups reveal detail for (a) Bujumbura, (b) Kigali, (c) Kampala, (d) Nairobi and (e) Dar Es Salaam.

The results of testing three population mapping procedures with different levels of input census data against the high resolution Kenya enumeration area census data (figure 1), are shown in figure 4. At every level of input census data, EApop3, which used both the satellite derived settlement map and Africover data, produced the most accurate population distribution map. Except for when national-level census counts were used, the simple areal weighting approach (EApop1) proved to be the second most accurate approach. While EApop3 was the approach that produced the lowest RMSEs overall, by district, results varied with both EApop1 and 2 proving more accurate approaches for certain districts (figure 5). Comparisons against Africover settlement polygons with population counts attached showed EApop3 to again be the most accurate approach, with a RMSE of 62430 people, in contrast to 80367 for EApop1 and 68172 for EApop2. Comparing the four existing gridded population products, APD, GRUMP, GPW3 and Landscan against the Kenyan enumeration area census data produced the overall RMSEs shown in table 2. GRUMP produced the lowest RMSE of the four, with 703.9 people, but none were able to improve upon the RMSE of 574 for EApop3. Finally, EApop3 was applied using the settlement map, Africover data and highest administrative level census data (figure 1) for the East African region. A three-dimensional representation of the resultant population density map is shown in figure 6.

**Table 2:** Details of the Kenya population maps produced, as well as existing products, and accuracy statistics calculated using the 43,733 EA census counts.

Surface	Administrative level of input census data	Ancillary data types used	Native spatial resolution	RMSE (no. people)	Standard error	Adjusted r^2^
EApop1	5	-	100 m	592.1475	530.64	0.608
EApop2	5	settlements	100 m	1097.754	971.51	0.365
EApop3	5	settlements, land cover	100 m	574.1875	509.7	0.625
APD	3	roads, rail, rivers, urban centers	2.5 minutes	1329.88	1298.07	0.276
GRUMP	5	urban extents	30 arc sec	703.9369	606.62	0.507
GPW3	5	-	2.5 minutes	1047.2101	904.44	0.411
LANDSCAN	3	roads, urban extents, elevation, slope, land cover	30 arc sec	1484.91	1365.19	0.232

## Discussion

For large regions of the World, spatially-referenced settlement and population data are outdated, of poor quality or lacking entirely. The results presented here show that it is possible to create detailed and accurate settlement and population maps of low income nations using cheap or freely available data. The most accurate approach involved a simple and easily updated methodology that required relatively few operators, and produced results that were substantially more accurate than existing datasets and at a much finer spatial resolution.

### Settlement mapping

The accuracies with which the Africover-defined test pixels were mapped (table 1) show that the settlements large enough to feature in the Africover land cover layer were well identified, their extents were relatively well mapped, and those areas defined as containing no settlements were also correctly mapped in general. The Africover production process leads to the creation of simple settlement outlines and definitions, which do not capture extent details or the variety of land covers within a settlement seen at 30 m spatial resolution, and this explains principally the discrepancies seen for the settlement statistics in table 1, particularly the relatively high settlement errors of omission. In terms of simply the percentages of test Africover settlement polygons containing predicted settlement pixels, therefore, the results were: Burundi 100%, Kenya 98.8%, Rwanda 100%, Tanzania 98% and Uganda 97.5%. Testing whether the smaller ‘settlements’ identified through the classification process were actually settlements, and were mapped accurately was also difficult, given the lack of data that exists for the EA region on such small settlements. For example, whilst Africover represents the most detailed datasource on settlements and their extents for the region, just 323 settlements are mapped for Kenya, whilst the process described here identified over 7000 distinct groups of ten or more contiguous settlement pixels. Such features mean that, whilst Africover does represent the best data available and accuracies were generally high, a full rigorous assessment of the accuracy with which small settlements were mapped in the region is unfeasible. Visual comparison between ‘settlements’ mapped and both Google Earth and the Landsat imagery (e.g. figure 3) does however suggest that the mapping accuracy of these smaller settlements was high across the region. Moreover, overlaying the comprehensive road network layer for Kenya identified that 81% of pixels classified as ‘settlement’ fell within 250 m of a road, suggesting correct mapping.

While the accuracies presented in table 1 are relatively high, difficulties within the settlement mapping process did lead to potential errors. Tatem et al [13] showed that the combination of imagery from passive and active satellite sensors, together with derived texture layers, produced the highest mapping accuracies. However, when mapping was undertaken with just radar or just Landsat imagery, the radar mapping produced much lower accuracies. Therefore, in those locations where the selection of cloudy Landsat imagery was unavoidable, settlement mapping accuracy is likely to be lower (especially in rugged areas), though sufficient verification data was unavailable to test this. Spectral confusion in a few small areas also contributed to potential settlement mapping errors, where what appeared to be non-settlement land cover had an almost identical spectral and radar response to settlements identified using Africover polygons. This occurred in only a small number of places, and was corrected where clear misclassification had occurred. Finally, in the cases where selection of neighbouring Landsat scenes acquired at different times of the year (Figure S1) were unavoidable, differing lighting conditions and vegetation growth stages are likely to have contributed to some spatial inconsistency within the settlement mapping [15].

### Population mapping

Hay et al [10] suggested that mapping accuracy improvement over simple areal weighting of census data could only be achieved with relevant ancillary data layers of finer detail, and a suitable modelling framework. Figure 4 provides evidence for this, with EApop3 producing small overall accuracy improvements over EApop1 through the use of detailed settlement information and a modelling approach that takes account of detailed land cover information. It is clear from the results of EApop2, that without the right modelling procedure, the use of high resolution ancillary data is no guarantee of mapping accuracy improvement over AW. Figure 4 also acts as a useful guide, demonstrating the accuracy levels and changes expected, given the administrative levels of census data available for mapping. It underlines the message that obtaining as high a resolution of census data as possible should be the priority starting point in map production, with the gradient of EApop1 and 3 indicating the improvements that can be made. Whilst figure 4 does demonstrate that EApop3 positioned populations more accurately than the other approaches overall, figure 5 shows that this was not achieved consistently for all Kenyan districts.

Table 2 emphasises further the importance of high resolution census data. Both GRUMP and GPW3 used sublocation (administrative level 5) data for Kenya, resulting in substantially lower RMSEs than APD and Landscan, each of which used level 3 data. Moreover, figure 4 shows that simple areal weighting of level 3 (division) data produced improved accuracy over APD and Landscan, indicating that the modelling approaches used were detrimental to mapping accuracy. With a RMSE lower than GRUMP and GPW3, however, the modelling approach of EApop3 proved more accurate, though the 1km and 5km resolutions of the original GRUMP and GPW3 mean that conclusive comparisons are difficult to make.

The question remains of whether further improvements in mapping accuracy over EApop3 can be made without significant additional costs or effort. The largest improvements in accuracy are likely to be achieved through the use of even higher administrative level census data. In the absence of this, the possibility of using additional ancillary data layers and rules should be considered. The phenomenon of human populations clustering around roads and other access routes has been exploited in the past [22], [23], though for much of the World, the data that exists on routes is incomplete [24], and the use of such data is likely to be detrimental to mapping accuracy (Table 2, [10]). Masks of zero population are another alternative, though aside from waterbodies (for which reliable and sufficiently high resolution data is rare), deciding what constitutes an area of no human habitation is difficult, with settlements existing around the world in national parks, industrial areas and deserts for instance.

The 100 m gridded population maps produced using EApop3 for the entire EA region, or individual countries within it, are freely available as a product of the Malaria Atlas Project (MAP: http://www.map.ox.ac.uk) and can be obtained by contacting Dr Andrew Tatem andy.tatemzoo.ox.ac.uk.

### Future Applications

The semi-automated approach that produced the most accurate maps here can easily incorporate new data, therefore, the maps outlined in this paper will be updated regularly as new census, satellite and ancillary data are released. The availability of improved datasets, such as census data at higher administrative levels or alternative satellite imagery, e.g. ASTER [25], are likely to also enable methodological and accuracy improvements. Numerous applications of the existing population maps are planned to exploit the increased spatial resolution, including the refinement of malaria risk and burden estimates [26], health system commodity estimation and medical facility accessibility modelling [18]. Satellite, census and land cover data stretch back to the 1970s, so the potential exists to also create high resolution gridded population maps for the past three decades. From such maps, valuable insights into the spatial patterns and processes that govern settlement development and population growth in low income regions could be gleaned that are not possible using existing spatial population databases, facilitating the modelling of future changes. Moreover, the substantial increase in detail of the maps over existing products potentially enables, for the first time for many countries, spatial epidemic model construction, high resolution poverty mapping and human movement modelling, amongst others.

Expansion of the Africover project (www.africover.org) to many other countries within Africa, under the FAO-Global Land Cover Network (www.glcn.org), and the initiation of similar projects in low income countries elsewhere, means that coincident expansion of high resolution population mapping is a possibility, with global Landsat imagery (GLCF: http://glcf.umiacs.umd.edu/, OnEarth: http://onearth.jpl.nasa.gov/) administrative boundaries (SALB: http://www.who.int/whosis/database/gis/salb/salb_home.htm, GADM: http://biogeo.berkeley.edu/gadm/, Statoids: http://www.statoids.com/statoids.html) and census data (GeoHive: http://www.geohive.com) freely available. The work described in this paper was undertaken with contributions from a team of just five people in three months. With settlements and populations mapped at 100 m resolution for almost 2 million km^2^ and the sole restricting expenses being software licenses and Radarsat data, the final production costs were just US$0.011 per km^2^ (excluding personnel costs). Advances in free image processing software (e.g. Multispec, http://cobweb.ecn.purdue.edu/biehl/MultiSpec/, GRASS, http://grass.itc.it/), the fact that relatively accurate mapping without radar imagery can be undertaken [13] and the increasing availability of free high quality and contemporary satellite and ancillary data, mean that mapping at below 1 cent per km^2^ should be feasible. The opportunity exists therefore to extend the approaches tested here towards a valuable and cost-effective Africa-wide settlements and population mapping project.

## Supporting Information

Text S1 Materials and Methods
(0.18 MB DOC)

Table S1 Average population densities for each adapted Africover class in Kenya, as defined by the Kenyan enumeration area census data.
(0.05 MB DOC)

Figure S1 The acquisition month of Landsat ETM imagery used in settlement mapping
(9.13 MB TIF)

Figure S2 Mosaic of Radarsat imagery used in settlement mapping.
(0.88 MB TIF)

Figure S3 Simplified 22-class Africover land cover classification used for population mapping.
(8.84 MB TIF)
