# Supplementary material for: High Resolution Population Maps for Low Income Nations: Combining Land Cover and Census in East Africa
Source: PLoS One. 2007 Dec 12;2(12):e1298. doi: 10.1371/journal.pone.0001298 (PMC2110897; doi:10.1371/journal.pone.0001298)
Supplement: Text S1 — Materials and Methods (0.18 MB DOC) [file pone.0001298.s001.doc]

**Supporting Information**

Materials and Methods

*Satellite Imagery*

Following the approaches outlined in Tatem et al [1,2], medium spatial resolution passive and active satellite imagery were obtained for the East African region covering an area of 1,817,955 km^2^. Radarsat-1 mosaics for each country were obtained from MDA Geospatial Services (Richmond, Canada), comprising of data collected between 1999 and 2002. The images were processed to extract texture information following Tatem et al [1,2].

Landsat Enhanced Thematic Mapper (ETM) scenes covering the East African region were acquired, free of charge, from the Global Land Cover Facility (GLCF, [http://glcf.umiacs.umd.edu](http://glcf.umiacs.umd.edu/)). To provide the best match to the most recent census data available for the region (see below), imagery from 2002, or as close to this year as possible were obtained. Imagery choice was further constrained by attempting to maintain between-scene temporal consistency [3] and minimising cloud cover and other detrimental atmospheric effects. Each image scene was subject to COST atmospheric correction [4,5] and georegistration was checked against all other imagery and ancillary data layers. Figure S1 shows the Landsat ETM tile extents, shaded by month of acquisition, while figure S2 shows the Radarsat mosaic for the EA region.

*Landcover and other data sources*

Full resolution Africover ([www.africover.org](http://www.africover.org/)) land cover data for the East Africa region countries were obtained. The 99 individual classes were aggregated to a more generic 22 classes to provide a consistent legend across the entire region. Figure S3 shows the resulting map. The Africover data uses Land Cover Classification software (LCCS) for creating standardized and harmonized land cover classes. This allows easy aggregation of land cover classes based on a hierarchy of land cover class detail, and allows comparison of land cover classes across countries and regions. In addition, Africover roads, rivers and towns products were obtained, along with other data on national parks, urban centres and health facilities to aid mapping, testing and accuracy assessment. The roads dataset was supplemented with various other datasets to ensure the inclusion of smaller unpaved roads [6].

*Census Data*

Human population census data and corresponding administrative unit boundaries from the most recent censuses in Burundi, Kenya, Rwanda, Tanzania and Uganda were obtained at the highest level available, and these are shown in figure 1 of the main paper. For Burundi, 1999 population data at administrative level 2 (commune) with corresponding boundaries were obtained through the Gridded Population of the World (GPW) project at Columbia University, New York (<http://sedac.ciesin.org/gpw/country.jsp?iso=BDI>). For Kenya, 1999 census data at administrative level 5 (sublocation) with corresponding boundaries were obtained from the Kenya National Bureau of Statistics (KNBS). Also obtained from the KNBS were Kenyan 1999 census data at enumeration area level (finer than level 5) with corresponding boundaries for 50 of the 69 Kenyan districts (figure 2, main paper). These were processed at the Kenyan Medical Research Institute (KEMRI) to ensure that population counts and administrative boundaries reconciled with the other levels of census data used. For Rwanda, 1991 population data at administrative level 2 (commune) with corresponding boundaries were also obtained through GPW (<http://sedac.ciesin.org/gpw/country.jsp?iso=RWA>). For Tanzania, 2002 census data at administrative level 4 (ward) with corresponding boundaries were obtained from the International Livestock Research Institute (ILRI) (<http://www.ilri.org/gis/search.asp?id=442>). For Uganda, 2002 census data at administrative level 4 (Parish) with corresponding boundaries were obtained from the Uganda Bureau of Statistics (UBS). Finally, to aid population map assessment, 1999 Kenya settlement population counts were obtained from the KNBS and matched to corresponding Africover ‘urban area’ or ‘rural settlement’ polygons.

*Gridded population products*

To enable brief comparisons of population maps produced using the approaches outlined in this paper and existing gridded population products, for Kenya the African Population Database (APD, <http://www.na.unep.net/globalpop/africa/>), the Global Rural-Urban Mapping Project (GRUMP, <http://sedac.ciesin.org/gpw/>), the Gridded Population of the World version 3 (GPW3, <http://sedac.ciesin.org/gpw/>) and Landscan 2005 (<http://www.ornl.gov/sci/landscan/>) were obtained.

*Settlement mapping*

Identification of settlements and the mapping of their extents were based upon the methodologies outlined in Tatem et al [1,2] and readers should refer to these for full details. A range of adaptations were made to the approaches, however, for simplification, ease of repetition and the incorporation of new data.

To ensure non-prohibitive processing time, ease of update and minimal information loss through mosaicing, settlement mapping was undertaken at the level of the Landsat tile extent (Figure S1). The Radarsat imagery and texture layers were therefore cut to match these extents. Whilst Radarsat data have proved valuable in improving settlement identification and mapping [1], in areas of great topographic variation, the radar responses due to the topography are often greater than, or mistaken for, those from settlements. Therefore, a ‘terrain ruggedness index’ image was created from a 90 metre spatial resolution Shuttle Radar Topography Mission (SRTM) digital elevation model (DEM), using the approach described by Riley et al [7] and those pixels with a value of 500 or greater (‘highly’ or ‘extremely’ rugged) were identified. For these pixels, Landsat ETM imagery alone was used in the settlement mapping procedure. A small number of areas were obscured by cloud within the Landsat imagery. The Radarsat imagery and its derived texture layers were used solely in classification, therefore, making the settlement mapping less precise for these areas [1].

Settlement types and reflectance characteristics are often dependent upon their setting and surrounding land cover. For the imagery in each tile extent, therefore, Africover data was used to identify land cover units (excluding settlement polygons) within which separate settlement mapping would take place. Accounting for the wide variation in reflectances within the generic land cover classes, the imagery representing each land cover type in a tile was then clustered into a conservative 1000 classes (thus reducing the possibility of spectral confusion through choosing too few classes) using ISODATA unsupervised classification [8].

Africover ‘urban area’ and ‘rural settlement’ class polygons (n=1318) formed the basis for training and testing of the settlement mapping. Within each Landsat tile extent, 75% of these polygons were chosen randomly for training, ensuring where possible that at least one ‘urban area’ and one ‘rural settlement’ polygon were within this selection, with the remainder set aside for accuracy assessment. For every tile, within each land cover region for which unsupervised classification had taken place, an example of ‘urban area’ and ‘rural settlement’ Africover polygons were identified from the training set, where possible. Within these polygons, image classes were highlighted and merged iteratively to best represent the training polygon extents, whilst discounting clear non-settlement land covers within the polygons, and produce a satellite imagery derived settlement map. This map was then examined briefly to identify and remove any unusual features produced through the spectral similarity of non-settlement land covers to settlements. The individual tile-level settlement maps were then mosaiced, and the overlap regions between tiles checked for consistency. Where conflict existed within these regions, the ancillary Africover layers and the original satellite imagery were checked to determine which classification decisions were most likely correct.

The 25% of Africover settlement polygons in each country that were set aside were used for accuracy assessment, as they represented the most reliable and consistent datasource on settlement presence and extent in the region. These polygons were rasterised to the same 30m spatial resolution grid as the settlement maps, and all grid squares within each settlement extent (188,321 grid squares in total) were identified for comparison with the settlement map and calculation of accuracy statistics. Following Tatem et al [1], an equal sized set of ‘non-settlement’ grid squares were randomly selected from non-settlement Africover classes to test whether the predicted settlement maps had identified false areas of settlement. Half of these grid squares were positioned randomly within 500m buffers of Africover settlement polygons to also assess the accuracy by which settlement extents were delineated by the maps. Percentage correct, Kappa and errors of commission and omission were calculated [9].

*Population mapping*

To match the most recent census data used and the majority of the satellite imagery, the population data for Burundi, Kenya and Rwanda were adjusted forward to estimated 2002 levels. This was undertaken with the equations outlined in Hay et al [10], using provincial inter-censal growth rates for Kenya and growth rates derived from National Statistical Office 1995 and 2000 population estimates for Burundi and Rwanda. To ensure consistency between countries and with global datasets, census data were clipped to the national boundaries defined by the second administrative level boundaries (SALB) project of the World Health Organization (<http://www3.who.int/whosis/gis/salb/salb_home.htm>). In some cases this involved the loss of parts of census units, therefore, their population totals were adjusted proportional to the area lost.

Three approaches to the creation of gridded population distribution maps from census data and/or the satellite imagery derived settlement maps were used.

- EApop1: the census data was simply areal-weighted (AW) [10] to a 100m spatial resolution grid, meaning that the sublocation population counts were equally distributed over the 100m pixels within each polygon.
- EApop2: the satellite derived settlement map for each country was degraded to the same 100m grid using a simple maximum filter. Where a settlement or multiple settlements were identified within an administrative unit, the population count was areal weighted over the settlement pixels. All other pixels in the unit were given a zero value. Where no settlements were identified within an administrative unit, the population count was simply areal weighted over the entire unit.
- EApop3: the satellite imagery derived settlement maps were degraded to 100m spatial resolution using a simple maximum filter, and ‘burned’ into the Africover land cover layers to create a refined land cover map for the region. Where settlement extent was mapped as smaller in the settlement map than the ‘urban area’ or ‘rural settlement’ classes in Africover, the surrounding land covers were grown to infill the gaps. This refined land cover layer and Kenyan enumeration area census data were then used to define per land cover class population densities (Table S1). These Africover-defined densities were then used as weights to distribute the census data across the entire region to create a population map. Though the land cover relationships were defined solely in Kenya due to the availability of fine resolution census data, this approach was justifiable as the country contains all the types of land cover and ecozone (Figure S4, below) found across the entire East Africa region.


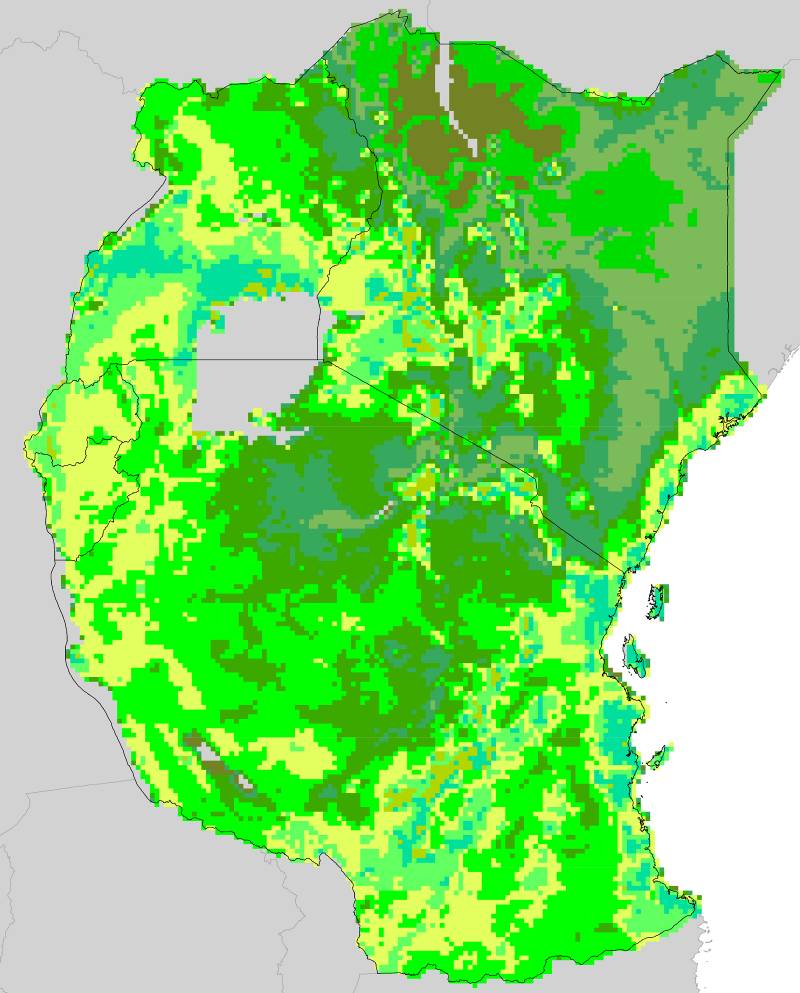


Figure S4. 10-class unsupervised clustering of AVHRR min, max and mean land surface temperature (LST), middle infrared reflectance (MIR) and normalised difference vegetation index (NDVI) to identify ‘ecozones’. All ten ecozone types identified for the EA region occur in Kenya.

The accuracies of the various population mapping procedures were principally tested using the enumeration area level census data for 50 Kenyan districts (figure 2, main paper). With a total of 43,733 enumeration areas and an average of 9 per sublocation, these provided an indication of the accuracy with which populations had been distributed within each sublocation. Additionally, to provide a finer resolution measure of settlement population mapping accuracy, the Africover settlement extents with assigned populations were used.

For each 100m gridded population distribution map produced using the differing approaches, the population numbers falling within each enumeration area and settlement polygon were extracted. These numbers were compared against the actual population figures with overall and district-specific root mean square errors (RMSEs) calculated. To explore the effectiveness of the population mapping procedures in the absence of high resolution census data, for Kenya the map production process was repeated using census data at administrative levels 4, 3, 2, 1 and 0 (national). The resulting maps were again compared against the enumeration area census data and RMSEs calculated. Finally, the maps of Kenya from existing gridded population products (APD, GRUMP, GPW3 and Landscan 2005) were adjusted to 2002 using the same growth rates described above, areal weighted to a 100m grid and compared to the enumeration area census data to obtain estimates of their accuracy relative to the approaches outlined in this paper.

*References*

1. Tatem AJ, Noor AM, Hay SI (2004) Defining approaches to settlement mapping for public health management in Kenya using medium spatial resolution satellite imagery. Remote Sensing of Environment 93: 42-52.

2. Tatem AJ, Noor AM, Hay SI (2005) Assessing the accuracy of satellite derived global and national urban maps in Kenya. Remote Sensing of Environment 96: 87-97.

3. Tatem AJ, Nayer A, Hay SI (2006) Scene selection and the use of NASA's global orthorectified Landsat dataset for land cover and land use change monitoring. International Journal of Remote Sensing 27: 3073-3078.

4. Chavez PS (1996) Image-based atmospheric correction - revisted and improved. Photogrammetric Engineering and Remote Sensing 62: 1025-1036.

5. Mahiney AS, Turner BJ (2007) A comparison of four common atmospheric correction methods. Photogrammetric Engineering and Remote Sensing 73: 361-368.

6. Noor AM, Gikandi PW, Hay SI, Muga RO, Snow RW (2004) Creating spatially defined databases for equitable health service planning in low-income countries: the example of Kenya. Acta Tropica 91: 239-251.

7. Riley SJ, DeGloria SD, Elliot R (1999) A terrain ruggedness index that quantifies topographic heterogeneity. Intermountain Journal of Sciences 5: 1-4.

8. ERDAS (2005) ERDAS Field Guide. Norcorss, Georgia: Leica Geosystems.

9. Campbell JB (2006) Introduction to Remote Sensing, fourth edition. New York: The Guilford Press.

10. Hay SI, Noor AM, Nelson A, Tatem AJ (2005) The accuracy of human population maps for public health application. Tropical Medicine and International Health 10: 1-14.
