# Supplementary material for: High Resolution Population Maps for Low Income Nations: Combining Land Cover and Census in East Africa
Source: PLoS One. 2007 Dec 12;2(12):e1298. doi: 10.1371/journal.pone.0001298 (PMC2110897; doi:10.1371/journal.pone.0001298)
Supplement: Table S1 — Average population densities for each adapted Africover class in Kenya, as defined by the Kenyan enumeration area census data. (0.05 MB DOC) [file pone.0001298.s002.doc]

| Africover class | Average population density (people per 100m^2^) |
| --- | --- |
| Airport | 0 |
| Artificial water body | 0 |
| Bare area | 0.10959 |
| Cultivated aquatic | 0 |
| Cultivated herb | 12.78433 |
| Cultivated shrub | 7.87330 |
| Cultivated tree | 23.81272 |
| Industrial area | 0 |
| Lake | 0 |
| Lake shore | 0.35456 |
| Natural aquatic | 0 |
| Natural herb | 6.45276 |
| Natural shrub | 4.49457 |
| Natural tree | 12.55062 |
| Quarry | 0 |
| Refugee camp | 0 |
| River | 0 |
| River bank | 0 |
| Rural settlement | 151.55820 |
| Sand beach | 0 |
| Snow | 0 |
| Urban area | 157.44890 |

Table 1. Average population densities for each adapted Africover class in Kenya, as defined by the Kenyan enumeration area census data.
