# Supplementary figures and images for: High Resolution Population Maps for Low Income Nations: Combining Land Cover and Census in East Africa
Source: PLoS One. 2007 Dec 12;2(12):e1298. doi: 10.1371/journal.pone.0001298 (PMC2110897; doi:10.1371/journal.pone.0001298)

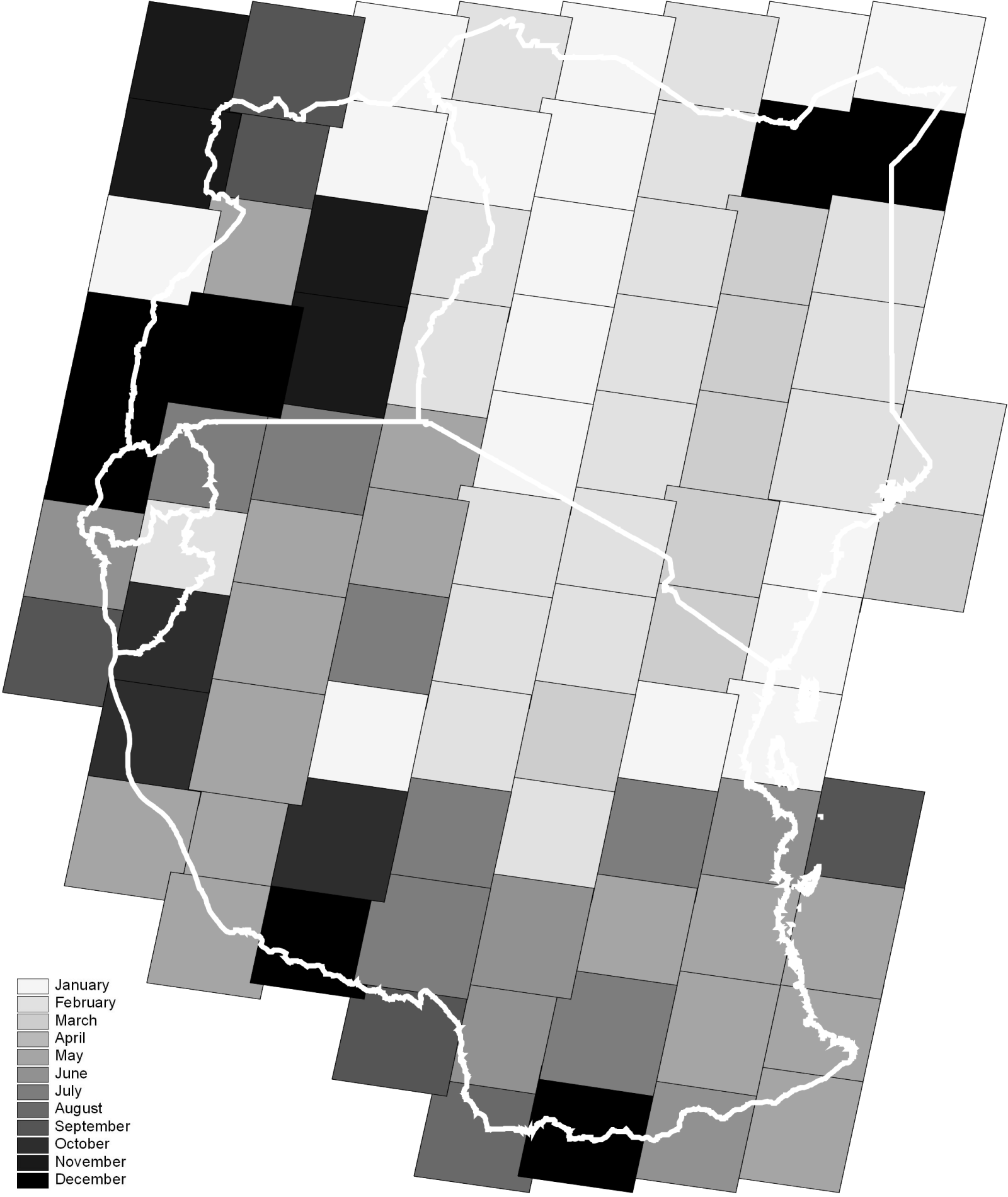

Supplement: Figure S1 — The acquisition month of Landsat ETM imagery used in settlement mapping (9.13 MB TIF) [file pone.0001298.s003.tif]

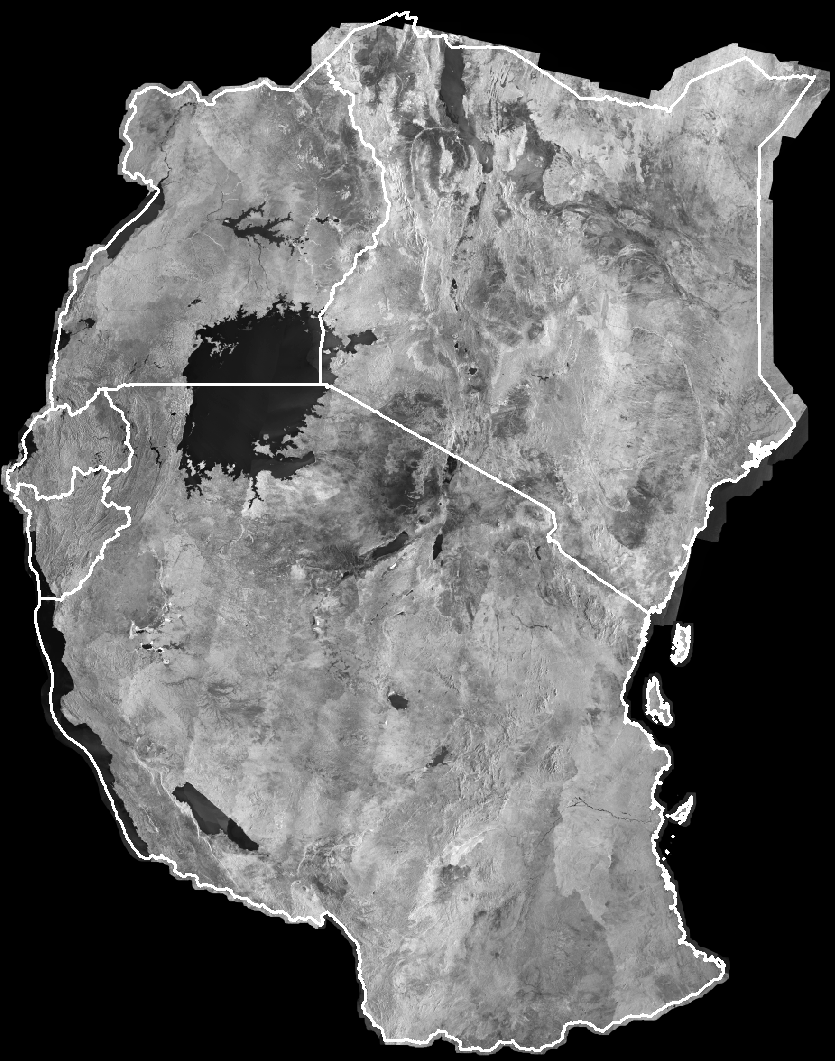

Supplement: Figure S2 — Mosaic of Radarsat imagery used in settlement mapping. (0.88 MB TIF) [file pone.0001298.s004.tif]

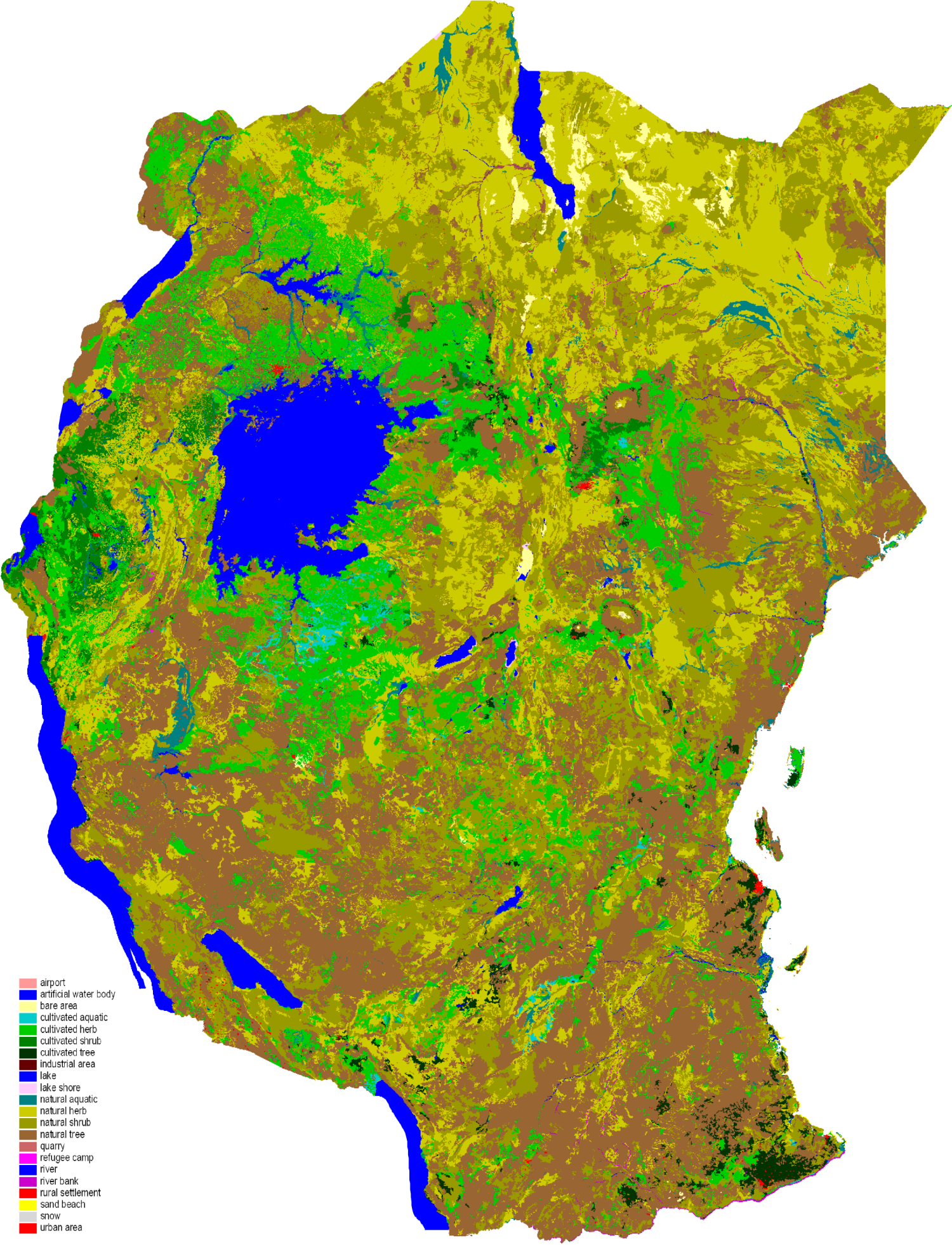

Supplement: Figure S3 — Simplified 22-class Africover land cover classification used for population mapping. (8.84 MB TIF) [file pone.0001298.s005.tif]
